# The infectious diseases clinical research program acute respiratory infection repository protocol: Opportunities to understand current and future epidemics

**DOI:** 10.1371/journal.pone.0317065

**Published:** 2025-07-23

**Authors:** Simon D. Pollett, Rhonda E. Colombo, Stephanie A. Richard, Tahaniyat Lalani, Brianne Barton, Allison Malloy, Anthony Fries, Edward Parmelee, Scott Merritt, Mark Fritschlanski, Edward E. Mitre, Eric D. Laing, Kathleen Pratt, Eric C. Garges, Katrin Mende, Mark Simons, Brian Agan, David Tribble, Robert O’Connell, Timothy H. Burgess

**Affiliations:** 1 Infectious Disease Clinical Research Program, Department of Preventive Medicine and Biostatistics, Uniformed Services University of the Health Sciences, Bethesda, Maryland, United States of America; 2 Henry M. Jackson Foundation for the Advancement of Military Medicine, Inc., Bethesda, Maryland, United States of America; 3 Madigan Army Medical Center, Tacoma, Washington, United States of America; 4 Department of Medicine, Uniformed Services University of the Health Sciences, Bethesda, Maryland, United States of America; 5 Naval Medical Center Portsmouth, Portsmouth, Virginia, United States of America; 6 Department of Pediatrics, Uniformed Services University of the Health Sciences, Bethesda, Maryland, United States of America; 7 US Air Force School of Aerospace Medicine, Dayton, Ohio, United States of America; 8 Department of Microbiology and Immunology, Uniformed Services University of the Health Sciences, Bethesda, Maryland, United States of America; 9 Department of Pathology, Uniformed Services University of the Health Sciences, Bethesda, Maryland, United States of America; 10 Brooke Army Medical Center, JBSA Fort Sam Houston, Houston, Texas, United States of America; Children's National Hospital, George Washington University, UNITED STATES OF AMERICA

## Abstract

**Background:**

Acute respiratory infections (ARI) are a major cause of morbidity and lost workdays in both military and non-military populations. To better understand these infections and their outcomes, the Infectious Diseases Clinical Research Program has enabled nine major ARI clinical research protocols in the last decade, including observational studies and trials, spanning emerging and reemerging ARI threats including Severe Acute Respiratory Syndrome Coronavirus 2, influenza, adenovirus, entero/rhinovirus, human metapneumovirus, respiratory syncytial virus, and other pathogens. These protocols have resulted in epidemiological, clinical and laboratory data and biospecimens from over 26,000 participants, most of whom were beneficiaries of a geographically distributed Military Health System.

**Methods:**

The Acute Respiratory Infection Repository Protocol establishes a unique Department of Defense (DoD) research resource through the pooling of data and specimens from nine ARI protocols into a master, standardized database with a linked specimen repository. This will enable further targeted scientific questions in participant-level pooled meta-analyses and will serve as an on-demand repository for rapid assay development, sample size estimations for prospective studies, and observational study/clinical trial design (including as part of future rapid pandemic research response). Accordingly, the objectives and study design of this protocol are broad. This protocol will allow analyses on outcomes including: (i) short-term ARI outcomes such as hospitalization, work days lost, symptom severity and duration; (ii) post-acute ARI outcomes, including persistence of symptoms, return-to-health, post-ARI medical encounters; (iii) vaccine effectiveness for Coronavirus disease 2019 (COVID-19), influenza, and adenovirus vaccines; (iv) ARI infection and vaccination elicited immune responses (humoral, T-cell, other); (v) therapeutic effectiveness of COVID-19 and influenza antivirals (acute symptoms, hospitalization, post-acute sequelae); (vi) effectiveness of non-pharmaceutical interventions (e.g., masking) against infection; (vii) prognostic and mechanistic host viral biomarkers which correlate with the above outcomes; (viii) ARI diagnostic assay validity and performance. This repository protocol is inherently broad in scope; the collation of standardized data and phenotype-linked specimens is a fundamental, primary objective.

**Discussion:**

This protocol will support statistical and laboratory analyses, including activities related to rapid epidemic response such as assay development and rapid sample size calculations for clinical trials. A series of more specific scientific questions from current and future collaborators will leverage this joint database and specimen repository; these questions will target important aspects of ARI infection, transmission, outcomes, and treatment. Future protocols (and ongoing data from existing IDCRP protocols) will be added to this collaborative repository protocol.

## Background

Acute respiratory infections (ARI) are a major cause of morbidity and lost duty days in military populations and remain a research priority for the U.S. military [1]. Acute respiratory infections pose a considerable risk to the health of all Military Health System (MHS) beneficiaries, and impact the readiness of active duty service members (ADSM). Acute respiratory infections may significantly impact military congregate populations in shipboard and fixed land-based facilities, including due to Severe Acute Respiratory Syndrome Coronavirus 2 (SARS-CoV-2), influenza, adenovirus, entero/rhinovirus, and other ARI viruses [1–3]. In military recruits and trainees, crowded living conditions, stress, and sustained exposure to ARI pathogens contribute to excess ARI risk [4–7]. Nearly a quarter of medical encounters among military recruit trainees were attributed to respiratory infections in both 2018 and 2019 [8,9]. Acute respiratory infections remained the second-leading cause of medical encounters in recruits in 2020 [10]. The 2020 SARS-CoV-2 outbreak on the aircraft carrier U.S.S. Theodore Roosevelt further illustrated how an ARI caused disruption in congregate shipboard settings [11]. Influenza has also caused substantive attack rates in Navy shipboard settings [12].

There remain multiple knowledge gaps about the epidemiology, optimal detection, prediction, treatment, and prevention of ARI in MHS beneficiaries and active duty servicemembers. Addressing these knowledge gaps is also beneficial to general public health. The overarching aim of this protocol is to establish a unique Department of Defense (DoD) research resource through the pooling of at least nine ARI protocols into a master, standardized database with a linked specimen repository. These ARI protocols have been executed under the auspices of the Infectious Disease Clinical Research Program (IDCRP), a clinical research center within the Department of Preventive Medicine and Biostatistics at the Uniformed Services University of the Health Sciences (USU) [13].

This repository protocol leverages prior extensive characterization of the epidemiology, clinical characteristics, virology, and immunology of COVID-19, influenza, and other respiratory viruses through multiple IDCRP ARI protocols. These protocols include (i) characterization of acute and chronic COVID-19 clinical outcomes in the Epidemiology, Immunology, and Clinical Characteristics of Emerging Infectious Diseases With Pandemic Potential (EPICC) Cohort Study [14,15]; (ii) a serocohort of military treatment facility (MTF) healthcare workers to characterize vaccine immunogenicity and correlates of protection (COP) against SARS-CoV-2 infections (the Prospective Assessment of SARS-CoV-2 Seroconversion [PASS] study) [16,17]; (iii) a SARS-CoV-2 congregate setting serocohort of Midshipmen at the US Naval Academy (The Observational Seroepidemiologic Study of COVID-19 at the United States Naval Academy [TOSCANA]) [18]; (iv) an assessment of SARS-CoV-2 infection risk in those deploying to a COVID-19 New York City field hospital [19], and (v) in those deploying to New York City on the COVID-19 USS Comfort response [20]; (vi) an assessment of COVID-19 transmission risk in house-hold units associated with Uniformed Services University faculty and students; (vii) a four-year influenza vaccine effectiveness study of three licensed products (the Pragmatic Assessment of Influenza Vaccine Effectiveness in the Department of Defense study [PAIVED] study) [21]; (viii) characterization of inpatient and outpatient ARI in the pre-pandemic era [22,23]; and (ix) evaluation of pH1N1 vaccine immunogenicity in those with and without immunosuppression [24].

The ARI Repository Protocol will provide a flexible platform for statistical and laboratory analyses, including activities related to rapid epidemic/pandemic response (e.g., assay development and rapid sample size calculations for clinical trials). Future IDCRP ARI protocols (and new data from ongoing IDCRP ARI protocols) will be added to this repository protocol which will remain open indefinitely. It should be noted that this repository protocol is inherently broad in scope and the collation of standardized data and phenotype-linked specimens is a fundamental, primary objective. The joint database and specimen repository can then be leveraged to answer a series of more specific scientific questions which will target important aspects of ARI infection, transmission, outcomes, treatment, and prevention to inform Force health protection and general public health.

## Methods/design

### Study population and eligibility criteria

Initially, this study involves the use of existing data and specimens from nine IDCRP ARI study protocols (Tables 1, 2, S1 Table). These protocols include: The Acute Respiratory Infection Consortium study (ARIC; IDCRP-045) [22,23]; the Immunogenicity of Novel H1N1 Vaccination among HIV-Infected Compared to HIV-Uninfected Persons (IDCRP-053) study [24]; the Epidemiology, Immunology and Clinical Characteristics of Emerging Infectious Diseases with Pandemic Potential study (EPICC, IDCRP-085) [14,15,25–39]); the Pragmatic Assessment of Influenza Vaccine Effectiveness in the DoD study (PAIVED, IDCRP-120) [40]; the COVID-19 Antibody Prevalence in Military Personnel Deployed to New York study (CAMP NYC, IDCRP-125; data only) [19]; the Prospective Assessment of SARS-CoV-2 Seroconversion study (PASS, IDCRP-126) [17,41,42]; the Seroprevalence of novel coronavirus antibodies among personnel deployed on the USNS Comfort during the COVID-19 pandemic study (COMFORT, IDCRP-128) [20]; The Observational Seroepidemiologic Study of COVID-19 at the United States Naval Academy study (TOSCANA, IDCRP-129) [43]; and the Prospective Investigation of SARS-CoV-2/ COVID-19 Epidemiology and Serology study (PISCES, IDCRP-130). Participant recruitment has been completed for all of these original studies, and results have been published from the original studies. Specimen laboratory data and/or additional longitudinal data is still being collected for the EPICC and PAIVED studies, and other protocols may be added to the ARI Repository Protocol in the future.

**Table 1:** Contributing IDCRP Acute Respiratory Infection Protocol Study Characteristics (up to 27,230 total participants).

Study (Protocol #)	Participant *n*	Pathogen(s)/Syndromes	Endpoints (data sources)	Specimen types (and associated lab data)
ARIC (IDCRP-045)	2,312	Influenza, entero/rhinovirus, hCoV, RSV, hPIV, hMPV, ILI	Infection, severity (CRF)	Swabs (multiplex PCR, sequence data)^a^
H1N1 Flu Vaccine (IDCRP-053)	132	Influenza	Infection, immunity (CRF, MDR)	Sera (ELISA, CD4 count, HIV RNA levels), urine
EPICC (IDCRP-085)	7,911	COVID-19, CLI, other respiratory pathogens including entero/rhinovirus, influenza, hCoV, RSV, hPIV, hMPV	Infection, symptoms, acute or chronic complications or severity, immunity, linked transmissions (CRF/questionnaire, MDR, prospective imaging)	Sera (IgG binding/neutralizing antibodies), plasma (proteomics), PBMC (immunoseq, CITE-seq, functional assays), swabs (viral load, sequence data)^a^, viral isolates (viral load, phenotype), PAXgene (RNAseq, host genome data), clinical residual specimens (viral load)^b^
PAIVED (IDCRP-120)	15,448	Influenza, COVID-19, CLI, ILI, entero/rhinovirus, hCoV, RSV, hPIV, hMPV	Infection (randomized trial primary endpoint), symptoms, immunity (CRF/questionnaire, MDR)	Sera (IgG binding/neutralizing antibodies), PBMC (functional assays), Swabs (viral load, sequence data)^a^, host DNA from cheek swab, saliva (IgG, IgA)
CAMP NYC (IDCRP-125)	372	COVID-19	Infection	Sera (IgG data only, no specimens)
PASS (IDCRP-126)	280	COVID-19, CLI	Infection, symptoms, acute severity, immunity (CRF/questionnaire)	Sera (IgG binding/neutralizing antibodies), PBMC (immunoseq, functional assays), saliva (IgG, IgA), clinical residual swabs
COMFORT (IDCRP- 128)	450	COVID-19, CLI	Infection (CRF/questionnaire, MDR)	Sera (IgG)
TOSCANA (IDCRP- 129)	196	COVID-19, CLI	Infection, immunity (CRF/questionnaire, MDR)	Sera (IgG), saliva (IgG, IgA)
PISCES (IDCRP-130)	129	COVID-19, CLI, influenza, entero/rhinovirus, hCoV, RSV, hPIV, hMPV hCoV, RSV, HPIV HMPV	Infection, immunity, linked transmissions (CRF/questionnaire)	Sera (IgG), swabs^a^, saliva

CLI = COVID-19-like illness; ILI = influenza-like illness, hCoV = non-SARS-CoV-2 endemic coronarviruses, RSV = respiratory syncytial virus, hPIV = parainfluenza viruses, hMPV = metapneumoviruses; PBMC = peripheral blood mononuclear cells; CRF = case report form; IgG = immunologobulin; MDR = Military Health System Data Repository; ^a^Swabs predominantly refer to upper respiratory swab specimens; ^b^Clinical residual specimens include upper respiratory swabs, bronchoalveolar lavage, stool and CSF.

**Table 2:** Contributing Protocol Objectives.

Protocol Objectives and Aims
**EPICC (IDCRP-085): Epidemiology, Immunology and Clinical Characteristics of Emerging Infectious Diseases with Pandemic Potential**
**For COVID-19/ SARS-CoV-2 infection, clinical and demographic data will be evaluated, along with laboratory findings as appropriate, to assess key endpoints of interest to include, but not limited to, the following:** • Incubation period • Duration of illness or symptoms • Duration of functional disability • Duration of shedding from respiratory and gastrointestinal tract Incidence and duration of viremia • Predictors of severe disease: clinical signs, symptoms, laboratory features; host factors, e.g., smoking, vaping, alcohol consumption, comorbid illness and conditions, occupational health factors influencing lung disease; host biomarkers; virologic (strain or virulence) attributes • Supportive management required: O_2_, HFNC, NIPPV, ventilation; hemodynamics • Complications: e.g., bacterial pneumonia; bacteremia; AKI; liver injury; CNS involvement, etc. • Outcomes: resolution of symptoms; return to pre-illness functional status; requirement for O_2_; development or exacerbation of reactive airway disease; pulmonary function; resolution of medical complications; new treatment requirements, e.g., bronchodilators; other sequelae • Development of immunity (i.e., correlates), kinetics, duration of antibody detection/protection including in response to a COVID-19 vaccine; evaluation of reinfection cases/risk; estimation of vaccine effectiveness • Relationship between acute illness (or pre-illness) HCoV titer and infection, outcome, virologic parameters • Costs of care, costs of duty days lost
**PAIVED (IDCRP-120): A Pragmatic Assessment of Influenza Vaccine Effectiveness in the DoD**
• **Specific Aim #1**: Comparison of the relative effectiveness (prevention of laboratory-confirmed influenza illness) of three types of licensed seasonal influenza vaccines. • **Specific Aim #2**: (Immunogenicity substudy) Determine whether cell-culture-based, egg-based and recombinant influenza vaccines give comparable HI and PVN titers to egg- and cell-matched vaccine antigens. An outcome of this objective is the potential to determine whether cell-culture-based vaccine antigens can provide broader coverage of circulating viruses than egg-based vaccine antigens. • **Specific Aim #3**: To determine if the impact of the influenza vaccine on disease burden and the attributable healthcare costs differs by product type. • **Specific Aim #4***:* To evaluate the association host single nucleotide polymorphisms (SNPs) and immune responses to the influenza vaccine, influenza acquisition and influenza severity. • **Specific Aim #5**: Assess the burden of COVID-19 and explore the inter-relationship between influenza and COVID-19.
**PASS (IDCRP-126): Prospective Assessment of SARS-CoV-2 Seroconversion**
• **Specific Aim #1**: Determine the frequency of asymptomatic and pauci-symptomatic SARS-CoV-2 infection in a cohort of healthcare workers. • **Specific Aim# 2**: Investigate the serological footprint of anti-S glycoprotein antibodies to HCoV-OC43, -HKU1, -229E, and -NL63, and determine whether pre-existing antibodies to these HCoVs are associated with altered COVID-19 disease course. • **Specific Aim #3**: Determine if pre-existing immune responses to seasonal coronaviruses affect magnitude and duration of antibody and T-cell responses to SARS-CoV-2 vaccination.
**ARIC (IDCRP-045): The Acute Respiratory Infection Consortium**
• **Primary Objective:** Develop a consortium of DoD research sites, which are capable of collecting detailed prospective clinical data and biologic samples from subjects with respiratory infections, particularly ILI, for analysis of the impact of these illnesses on our active-duty members and their families. • **Secondary Objective A:** Develop and validate an influenza severity scale. A preliminary severity scale for ILI will be developed and applied. The scale will be based on subjective and objective findings, which are easily measured and duplicated (as many numeric scales as is practical). • **Secondary Objective B:** Study the relationship between health and fitness of young adults to outcomes of influenza and other respiratory pathogens. • **Secondary Objective C:** Examine the relationship of cell-mediated and humoral immune response to the severity of influenza and other respiratory pathogens. • **Secondary Objective D:** Describe patterns of viral shedding in different influenza types and subtypes. Specifically, describe the frequency of asymptomatic shedding, identify possible transmission associated with asymptomatic shedding, and describe the duration of shedding in subjects with different influenza viruses and across a wide spectrum of clinical disease. • **Secondary Objective E:** Correlate clinical severity and cytokine/humoral response with underlying host genotype. • **Secondary Objective F:** To determine the representativeness of the cohort to the referent population with regards to demographics, ILI/SARI severity, and pathogen distribution. • **Secondary Objective G:** To investigate interaction between influenza and other respiratory pathogens. • **Secondary Objective H:** To describe the pathogens associated with cases requiring intensive care or mechanical ventilation. Secondary Objective I: To study the use of antimicrobial agents in the treatment of ILI and SARI. • **Secondary Objective J:** To study inflammatory immune responses to pathogens associated with SARI. • **Secondary Objective K:** To characterize antimicrobial sensitivity patterns of ILI and SARI pathogens. • **Secondary Objective L:** To estimate influenza vaccine and adenovirus vaccine effectiveness. • **Secondary Objective M:** To explore ILI- and SARI-associated health care utilization and economic burden, duty and work absences associated with ILI and SARI. • **Secondary Objective N:** To examine site of care decisions (inpatient vs. outpatient) related to SARI and ILI.
**COMFORT (IDCRP-128): Seroprevalence of novel coronavirus antibodies among personnel deployed on the USNS Comfort and Mercy during the COVID 19 pandemic**
• **Primary Objective:** Determine the seroprevalence of COVID-19 antibodies among US Navy personnel deployed to Los Angeles or New York City on the USNS MERCY and COMFORT hospital ships in support of COVID-19 outbreaks in those cities. • **Secondary objectives:** Compare the seroprevalence of COVID-19 antibodies between the Navy personnel deployed on the USNS MERCY and those on the COMFORT. Correlate the likelihood of COVID-19 antibody prevalence with assigned duties (nonclinical, medic, nursing, physician), workspace, social circles, berthing, and known exposure to COVID-19 infected patients. Determine the incidence of medically-attended, laboratory-confirmed COVID-19 among enrolled personnel using medical record abstraction 1-year post-enrollment. Compare post-deployment seroprevalence to a convenience sample of those crew members with available DoD serum repository samples within 1–2 months prior to deployment.
**PISCES (IDCRP-130): Prospective Investigation of SARS-CoV-2/ COVID-19 Epidemiology and Serology**
• **Specific Aims:** To determine the prevalence and incidence of SARS-CoV-2 infection. To evaluate the association between baseline SARS-CoV-2 antibody seroprevalence and attack rate of SARS-CoV-2 over the course of a respiratory disease season. To describe the association between occupational-, household-, and community-level risk factors and the risk of SARS-CoV-2 infection. To describe the functional impact of SARS-CoV-2 and non-SARS-CoV-2 respiratory pathogens on the health of the USU community across one respiratory disease season. To characterize the elicitation and durability of the host immune response to SARS-CoV-2 and the influence of symptom/disease severity on the magnitude of the response. To examine the correlations between systemic and mucosal immune response following exposure to infection with SARS-CoV-2.
**TOSCANA (IDCRP-129): The Observational Seroepidemiologic Study of COVID-19 at the United States Naval Academy**
• **Specific Aims:** To determine the prevalence and incidence of SARS-CoV-2 infection among students at a military service academy. To evaluate the association between baseline SARS-CoV-2 antibody seroprevalence and attack rate of SARS-CoV-2. To evaluate the association between baseline SARS-CoV-2 antibody seroprevalence and rate of medically-attended ARI due to SARS-CoV-2. To describe the proportion and temporal distribution of medically-attended ARI due to SARS-CoV-2 relative to that of common seasonal respiratory pathogens. To describe individual-level risk factors (i.e., host genetic determinants) associated with progression to clinically-significant COVID-19. To evaluate COVID-19 mitigation approaches and the relative impact of these strategies on rates of all-cause as well as SARS-CoV-2 medically-attended ARI in a university dormitory setting.
**H1N1 Vaccine (IDCRP-053): Immunogenicity of Novel H1N1 Vaccination among HIV-Infected Compared to HIV-Uninfected Persons**
• **Primary Objective:** To compare the immunogenicity via anti-hemagglutinin responses following H1N1 vaccination between HIV positive and negative persons. Null Hypothesis (H_o_): The immunogenicity of the H1N1 vaccine by anti-hemagglutinin seroresponse between these groups will not be significantly different. • **Secondary Objectives:** (i) To compare the immunogenicity via HI titer levels, microneutralization seroresponses and titer levels, and cellular responses following H1N1 vaccination (i.e., visit 1 to visit 2 changes) between HIV positive and negative persons. Null Hypothesis (H_o_): The immunogenicity of the H1N1 vaccine by HAI titer levels, microneutralization seroresponse and cellular responses between these groups will not be significantly different. (ii) Among those undergoing vaccination with the seasonal influenza vaccine during the current influenza season, to compare the presence of a positive seroresponse between HIV positive and negative persons. H_o_: The presence of a seroresponse to seasonal influenza vaccination between these groups will not be significantly different. (iii) To evaluate the effect of pre-existing anti-influenza immunity and recent history of seasonal influenza vaccination on seroresponses to the H1N1 influenza vaccine among both HIV positive and negative persons. H_o_: Prior anti-influenza immunity or recent seasonal influenza vaccination will have no impact on H1N1 vaccine immune responses. (iv) To compare the durability of the H1N1 immunologic responses at 6 months post-vaccination between HIV-infected and uninfected persons. H_o_: The durability of the immune responses to the H1N1 vaccine as measured by antibody seroresponses and cellular responses between these groups will not be significantly different. (v) To evaluate the number of ILIs and documented influenza cases among HIV-infected and uninfected persons after initial vaccination, and to genetically characterize the influenza strains causing ILI events in our study cohort.
**CAMP NYC (IDCRP-125): COVID-19 Antibody Prevalence in Military Personnel Deployed to New York (Determination of novel coronavirus antibody prevalence among personnel in deployed in response to the New York City COVID-19 pandemic)**
• **Primary Objective**: To determine the seroprevalence of COVID-19 antibodies among active-duty Army personnel deployed to New York City to respond to the COVID-19 pandemic prior to their redeployment. • **Secondary objectives**: (i) To correlate the likelihood of COVID-19 antibody prevalence with frequency of exposure to COVID-19 infected patients. (ii) To correlate the likelihood of COVID-19 antibody prevalence with type of duties (nonclinical, medic, nursing, physician). (iii) To correlate the likelihood of COVID-19 antibody prevalence with history of performance aerosolizing procedures. (iv) To correlate the presence COVID-19 antibody prevalence with history of COVID-like symptoms. (v) To evaluate the performance characteristics of Premier Biotechnology qualitative COVID-19 antibody test. (vi) To determine the rate of viral RNA detection in patients with confirmed COVID-19 and correlate with the presence and titer of serum antibodies.

CNS = central nervous system; HFNC = high-flow nasal cannula; NIPPV = nasal intermittent positive pressure ventilation; AKI = acute kidney injury; HCoV = human coronavirus; HI = hemagglutinin inhibition; PVN = pseudovirus neutralization; DoD = Department of Defense; ILI = influenza-like illness; SARI = severe acute respiratory infection.

All studies included in the repository protocol have been conducted at military treatment facilities, in military training settings, within other DoD facilities, and/or in the context of DoD deployments related to the COVID-19 response. The overall population is therefore predominantly MHS beneficiaries, including (ADSM), retirees, and/or dependents. The nine studies included in this Repository protocol have had varying eligibility criteria (see S1 Table for inclusion and exclusion criteria) but generally fall under overarching repository eligibility criteria of MHS beneficiaries, including ADSM and those in congregate settings, who are infected by, exposed to, tested for, and/or vaccinated against ARI pathogens.

The majority of these studies, to date, are derived from a portfolio of studies established as part of COVID-19 research response. The EPICC study enrolled MHS beneficiaries 2020−2022 and with broad objectives to understand the epidemiology, immunology and clinical characteristics of COVID-19. This study has published multiple insights into the risk and risk factors of acute and long term complications of SARS-CoV-2 [14,28,44,45], COVID-19 clinical characteristics and symptomology [32], COVID-19 functional impact [15], COVID-19 diagnosis [46], SARS-CoV-2 immunology and vaccinology [33], and SARS-CoV-2 virology [35,47]. Data and specimens have been analyzed since March 2020, through to the present.

The PASS study examined SARS-CoV-2 seroconversions in a cohort of WRNMMC healthcare workers, in addition to licensed COVID-19 vaccine responses [42]. Enrollment commenced in August 2020 and last follow up occurred in June 2024. Data and specimens have been analyzed since August 2020 through the present. Analyses have included patterns and predictors of humoral, innate, and T cell immunity, including correlate of protection and variant escape analyses. Additionally, the PASS study has undertaken profiling of vaccine reactogenicity and COVID-19 symptomology across multiple years of the pandemic [17,34,41,48,49].

Two of the IDCRP COVID-19 studies focused on congregate settings or household transmissions. The TOSCANA study commenced enrollment of U.S Naval Academy midshipmen in 8/2020 with follow up through to 5/2021. TOSCANA examined the risk and risk factors of infection of SARS-CoV-2 in this congregate setting, including examining the ascertainment of infection by seroconversion compared to frequent population PCR testing [18]. Data and specimens were analyzed between 11/2021–1/2023. The PISCES study enrolled Uniformed Services University students and faculty, in addition to their respective households, to understand the risk and risk factors of SARS-CoV-2 infection and household transmission dynamics. Enrollment commenced in 2021/01/14 and concluded in 2021/06/17. Data and specimens were analyzed between 2021 and 2022.

The CAMP NYC enrolled military members who participated in the 2020 New York City Javitz Center COVID-19 clinical response. The study objectives focused on the risk and risk factors of SARS-CoV-2 infections (including infections ascertained by serology) and concluded that such risks were low in the context of high adherence to PPE [19]. Participants were enrolled in the CAMP-NYC study between 2020/03/24 and 2020/04/28; data and specimens were analyzed in 2020 and 2021. As similar conclusion was made with the COMFORT study which examined the seroepidemiology and SARS-CoV-2 infection risk in military members who deployed to New York City as part of the U.S.S Comfort COVID-19 medical response [19]. Participants were enrolled in the COMFORT study between 2020/05/09 and 2020/06/23; data and specimens were analyzed in 2020 and 2021

The PAIVED study was a large open-label Phase IV study of three licensed influenza vaccines (egg-derived inactivated, cell-derived inactivated influenza, and recombinant hemagglutinin) among MHS beneficiaries, enrolling between 11/7/2018 through 5/26/22. The primary objective was to examine clinical effectiveness of the three licensed vaccines, with multiple secondary immunological analysis and other objectives [21,40,50]. Data and specimens were analyzed from 2019 to present. Findings from the PAIVED study, to date, include (i) no major differences in vaccine effectiveness between egg-derived, cell-derived and recombinant-hemagglutinin influenza vaccines in the study sample [50]; (ii) similar A/H3N2 immunity elicited from egg and cell-based vaccines [40], and yet (iii) differential neuraminidase antibody responses elicited by egg and cell based vaccines [21].

The H1N1 vaccination study examined the immune responses to the pandemic H1N1 vaccine in those with and without HIV [51]. Participants were enrolled in the H1N1 study between 2009/11/09 and 2010/06/01; data and specimens were analyzed in 2010 through to 2017. This study contributed to our understanding of influenza vaccine immunogenicity between those with and without HIV infection, in addition to the role of vitamin D deficiency in H1N1 vaccine responses [24,51].

Finally, the ARIC study characterized the clinical outcomes and epidemiology of ARI across a multi-site cohort. Participants were enrolled in the H1N1 study between 2009/10/09 and 2019/04/12; data and specimens were analyzed from 2009 through to 2021. The ARIC study led to several insights into ARI in the MHS, including (i) similar influenza-like illness symptom severity and infection outcomes in those participants with and without HIV [23]; (ii) species, type and/or lineage-specific clinical phenotyping of influenza viruses, adenoviruses, rhinoviruses, and human coronaviruses [22,52–55]; (iii) patterns of influenza antiviral use [56] and; (iv) validation of quantitative symptom-scoring tool for influenza and other ARI [57,58].

### Recruitment and consent

The IDCRP ARI Repository Protocol does not recruit or enroll new subjects. Data and specimens will only be used from participants who provided consent for secondary use of data and/or specimens, and who did not withdraw from, the original studies contributing to the repository.

### Primary objective

The primary goal of the repository protocol is to create a curated, standardized dataset and biospecimen repository incorporating multiple IDCRP ARI protocols. This will permit answering further targeted scientific questions in pooled analyses and will serve as an on-demand repository for rapid assay development, sample size calculations, and observational study/clinical trial design (including those required as part of future rapid ARI pandemic research response).

### Secondary objectives (supported and facilitated through the Primary Objective)

1. Compare the acute and chronic clinical outcomes and symptom phenotypes of ARI viral pathogens of importance to servicemembers and other MHS beneficiaries, including variant to variant comparison
2. Estimate predictors of acute and chronic clinical outcomes of ARI viral pathogens, including demographics, comorbidities, other host factors, and viral factors including infecting variant
3. Estimate and predict COVID-19, influenza, and adenovirus vaccine effectiveness in servicemembers and other MHS beneficiaries, as applicable
4. Identify predictors of optimal ARI vaccine immune responses (including those elicited by COVID-19 and influenza vaccines)
5. Estimate therapeutic effectiveness for licensed COVID-19 and influenza antivirals
6. Estimate effectiveness of non-pharmaceutical interventions, including masking and restriction of movement, against infection risk
7. Identify and evaluate prognostic and mechanistic viral and host biomarkers which correlate with the above secondary objectives
8. Evaluate the validity of current and future diagnostic and biomarker assays for the detection or prognostication of ARIs, including the support of new ARI diagnostic and biomarker assay development
9. Additional objectives as urgent DoD ARI research needs arise (e.g., during an outbreak or pandemic)

### Sample size

Current sample size is up to 27,230 participants from at least nine existing IDCRP ARI Protocols (see Table 1). The sample size is fixed with the number of enrolled and eligible participants in the protocols being pooled. The observable effect sizes will vary by endpoints of specific pooled analyses (see Secondary Objectives) that will leverage this repository protocol.

### Data curation

**Fig 1:**
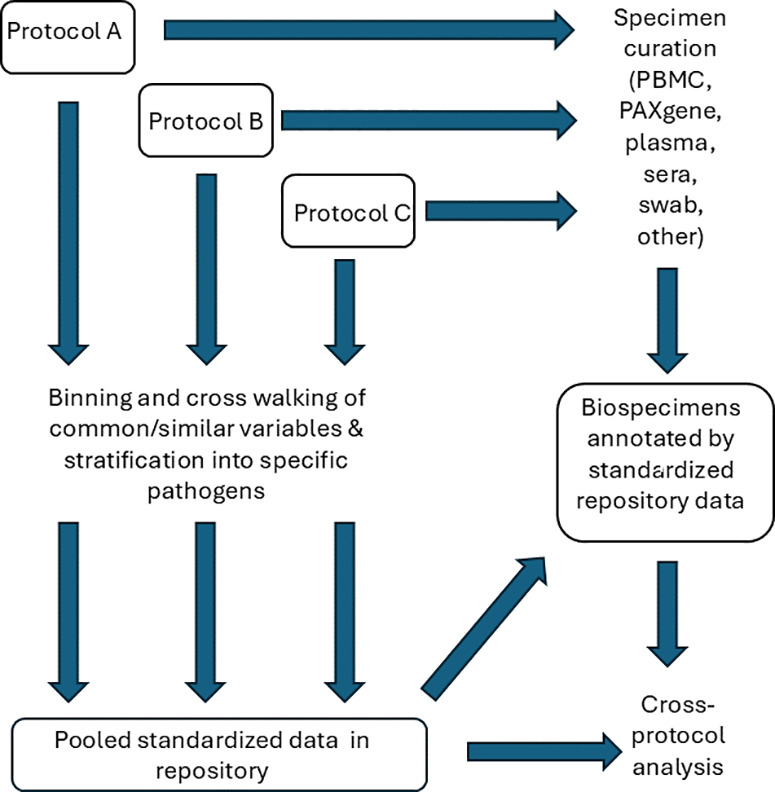
Schema showing the approach to data and specimen curation. Protocols A, B, and C are notional illustrative protocols.

The repository protocol will only contain data and specimens from participants who approved future use of data and specimens in their consent. Data will be combined into a unified database, utilizing existing and newly created variables (overview shown in Fig 1). A Data Management Plan (DMP) and cross protocol data dictionary will be established, ensuring data and specimens are only included in those participants who indicated consent for future use (or for studies with a waiver of consent). This multi-relational database will be developed in Oracle and managed by the IDCRP Data Coordination Center (DCC) and will include clinical, demographic, and existing laboratory data.

Following data consolidation, a statistical analysis plan (SAP) will be developed for a preliminary assessment of the repository data to evaluate metadata structure including the total number of combined variables (and whether these are continuous, binary, or categorical), total number of combined observations, total number of common data variables (and respective number of observations) across two or more protocols, and total number of variables (and respective number of observations) which are similar enough to allow ontological cross-walking between two or more protocols. The overlap of laboratory assay data between protocols will also be evaluated, with assay type, performing laboratory, lab assay units, and measurement timepoints specified. Similarly, we will evaluate the overlap of similar specimen types (e.g., sera and PBMC) and timepoints of collection between protocols.

Documentation of these newly created common variables in the cross-protocol data dictionary will include: (i) common repository variable name, (ii) data type (e.g., continuous, categorical), (iii) any variable ontology cross-walking between protocols (iv) reference to the original variable names from individual protocols. The joint repository dataset will include a variable which defines the original IDCRP study in which the participant enrolled (the study population and eligibility criteria of which are summarized in S1 Table). This will provide context of how individual participants were selected for enrollment and therefore identify potential selection biases in cross protocol analyses. As other open protocols are updated with further specimens and data, data will also be updated in the master repository protocol.

### Quality management

A Quality Management Plan (QMP, including a data Quality Assurance (QA)/Quality Control (QC) process) will be applied to ensure the accuracy, completeness, and acceptability of data/specimens from the contributing protocols. The QMP leverages data system checks, previously performed QM activities, and limited review of key protocol activities (informed Consent, data migration, and regulatory compliance). QM processes are designed to evolve during the lifecycle of this repository protocol, to continually mitigate previously- and newly identified risks.

### Specimen curation

An inventory of all specimens will be created and verified (overview in Fig 1), with cross-linkage to FreezerWorks a specimen freezer inventory program (Dataworks Development, Inc; Mountlake Terrace, WA). An initial report will describe the demographic, clinical and laboratory characteristics, and specimen availability. Data and specimens will be updated in the master repository protocol as other open protocols are updated with further specimens and data.

### Approach to analyses

After the scope and overlap of the data and specimens have been assessed, the consolidated data and/or specimens will be assessed to answer specific research questions as described in the secondary objectives with individual participant data and/or specimen results. In addition, the report will be used by the analysis team and collaborative investigators to help design new specific, hypothesis-driven questions that will be answered leveraging the repository protocol pooled data.

New analysis ideas will be vetted by an analysis working group (reflected by the byline authors of this paper). This is a collaborative framework in which (i) contributing principal investigators (PIs) have oversight on the use of specimens and data from their protocols and can be part of the joint scientific analysis enterprise, (ii) competing laboratory demands for specimens can be balanced early and often, (iii) lab and statistical analyses can be prioritized based on a consensus regarding the most impactful, specific study questions. New analysis ideas will be presented to the working group, and after consensus is reached, analyses will proceed if they are covered under the scope of the existing protocol.

The approach to individual participant-level data (IPD) pooled meta-analyses (secondary objectives) will be tailored to the specific scientific question. This will follow IPD analysis best-practices as described by Riley et al. [59] and IPD meta-analysis best practices for reporting results (further described in Riley et al [59]). Importantly, such ARI Repository IPD metanalyses will not necessarily occur across all contributing protocols. Consideration of the underlying sampled population, study design, and common variables will be incorporated into a DMP and SAP for each specific IPD metanalysis. The DMP and SAP for each IPD meta-analysis will include: analysis objectives (primary, secondary); analytic sample inclusion and exclusion criteria; dependent and independent variable definitions; approaches to poorly or incongruently defined endpoints and other variables across studies; assessment of dependent and independent variable normality and functional transformation as required for advanced analysis; effect size (e.g., risk ratio, odds ratio); causal inference approaches, including model fitting, model diagnostics and approach to repeated measures; approach to missing data; evaluation of patterns of missingness; subgroup analyses; and evaluation of effect modifiers. Specific statistical analysis approaches would be tailored to the study question, but may include linear regression, logistic regression, Cox-proportional hazard models, and mixed effects models.

Note that objectives such as *in-vitro* assay development and evaluation may be performed off relatively small number of biospecimens (in contrast to objectives which seek to evaluate sensitivity, specificity, likelihood ratios of established assays) [60,61].

### Generation of additional laboratory data from the specimen repository

While no new enrollment or participant data collection occurs in this repository protocol, secondary laboratory data, aligned with the secondary objectives listed, may be generated by current and future laboratory science collaborators using the specimens reposed in the ARI repository protocol. These may include host response analyses (e.g., [16,29,45,62,63]), virological and viral genomic analyses (e.g., [47]), and diagnostic evaluation (e.g., [35,39,46]).

### Ethics approval and consent to participate

Provisions related to future use of data and specimens for research purposes were included in the Informed Consent Documents and text of the original protocols being merged for this repository (with the stipulation that the future use would be under a protocol reviewed by the USU IRB). This ARI Repository Protocol (IDCRP-142) was approved by USU Institutional Review Board and originating protocols have had modifications which refer to the movement of data and specimens into IDCRP-142.

## Discussion

This ARI Repository Protocol is expected to directly support further pandemic research response protocols, as well as follow-on lab-based studies (e.g., mechanistic studies for optimal vaccine response). The intended uses for this joint repository protocol include rapid assay development for emerging acute respiratory epidemic and pandemic pathogens. An illustration of the importance of having ‘on-demand’ biospecimen access was the use of the IDCRP ARIC protocol specimens [22] to rapidly support a high-throughput SARS-CoV-2 assay developed by USU investigators. This assay was then used across multiple IDCRP and non-IDCRP COVID-19 protocols to support multiple high tier publications and further diagnostic assay development [33,48,49].

In addition, this ARI Repository Protocol will allow rapid sample size calculations to inform new studies in MHS populations (for example, an interventional clinical trial or a diagnostic assay validation study), comparator population analyses (for example comparing the morbidity of a new pandemic respiratory virus against COVID-19 or seasonal influenza), and cross-validation of prognostic biomarkers. Furthermore, this repository protocol will permit IPD meta-analyses to answer ARI questions with greater statistical power than individual studies can permit alone. These IPD analyses will be responsive to new respiratory virus epidemics and/or evolving MHS beneficiary health needs.

A strength of this ARI Repository Protocol is that many variables across the contributing protocols were measured in similar ways; for example, several studies used commonly worded CRF or survey response fields (e.g., FLUPRO scores across influenza and SARS-CoV-2 protocols [32,36,57]) and assays (e.g., common serology panels measured in the same USU lab across multiple COVID-19 protocols [18–20,33,48,49]. Nevertheless, there will be differences in measurement definitions and cross-walking approaches are not expected to be able to entirely standardize variable use across all protocols. Other limitations include differences in study populations (for example, some studies recruited across all ages, others recruited adult active duty only – see Table 1 and S1 Table), although recruitment for enrollment within the same MHS would be expected to at least partially mitigate this limitation. Other threats to internal validity when performing IPD analyses across two or more protocols within this repository include differences in enrollment approaches and study designs, which may introduce selection bias. Specific cross-protocol analyses leveraging this protocol will need to identify, mitigate (to the extent possible) and declare such limitations.

The scientific objectives for this ARI Repository Protocol have clear military relevance to diseases of significant ADSM impact. The publication history of the contributing individual IDCRP protocols underscores the military relevance of this joint ARI Repository Protocol effort. In addition, this ARI Repository Protocol will help support future ARI pandemic research response. New laboratories interested in partnering on ARI investigations are welcome to inquire about collaborative opportunities aligned with the scientific aims of this biorepository.

## Conclusions

This protocol will support statistical and laboratory analyses, including activities related to rapid epidemic response such as assay development and rapid sample size calculations for clinical trials. A series of more specific scientific questions from current and future collaborators will leverage this joint database and specimen repository; these questions will target important aspects of ARI infection, transmission, outcomes, and treatment. Future protocols (and ongoing data from existing IDCRP protocols) will be added to this collaborative repository protocol.

### Disclaimer

Views expressed are those of the authors and do not reflect the official policy of the Uniformed Services University of the Health Sciences, the Department of the Army, the Department of the Navy, the Department of the Air Force, the Department of Defense, the Defense Health Agency, or the U.S. Government and the Henry M. Jackson Foundation for the Advancement of Military Medicine, Inc. (HJF). The investigators have adhered to the policies for the protection of human subjects as prescribed in 45 CFR 46.

E.D.L, D.R.T, M.P.S, A.M, A.F, K.P, E.C.G, K.M, R.O., T.H.B, and E.M are U.S. Government employees or military service members. This work was prepared as part of the author(s) official duties. Title 17 U.S.C. § 105 provides that ‘Copyright protection under this title is not available for any work of the United States Government.’ Title 17 U.S.C. §101 defines U.S. Government work as work prepared by a military service member or employee of the U.S. Government as part of that person’s official duties.

## Supporting information

S1 Table IDCRP Acute Respiratory Infection Study Participant Information. (DOCX)

## Abbreviations

ADSM: Active duty service member
ARI: Acute respiratory infection
ARIC: Acute Respiratory Infection Consortium
CAMP NYC: COVID-19 Antibody Prevalence in Military Personnel Deployed to New York
COVID-19: Coronavirus disease 2019
CRF: Case report form
DCC: Data; oordination Center
DMP: Data Management Plan
DoD: U.S. Department of Defense
EPICC: Epidemiology, Immunology and Clinical Characteristics of Emerging Infectious Diseases with Pandemic Potential
FLUPRO: Influenza Patient-Reported Outcome
HIV: Human immunodeficiency virus
HJF: Henry M. Jackson Foundation for the Advancement of Military Medicine, Inc.
IDCRP: Infectious Disease Clinical Research Program
IPD: Individual participant-level data
IRB: Institutional Review Board
MHS: Military Health System
PAIVED: A Pragmatic Assessment of Influenza Vaccine Effectiveness in the Department of Defense
PASS: Prospective Assessment of SARS-CoV-2 Seroconversion
PBMC: Peripheral blood mononuclear cell
PI: Principal investigator
PISCES: Prospective Investigation of SARS-CoV-2/COVID-19 Epidemiology and Serology
QA: Quality Assurance
QC: Quality Control
QM: Quality Management
QMP: Quality Management Plan
SAP: Statistical analysis plan
SARS-CoV-2: Severe acute respiratory syndrome coronavirus 2
TOSCANA: The Observational Seroepidemiologic Study of COVID-19 at the United States Naval Academy
U.S.C.: United States Code
USNS: United States Navy Ship
U.S.S.: United States Ship
USU: Uniformed Services University of the Health Sciences.
