# Supplementary material for: The infectious diseases clinical research program acute respiratory infection repository protocol: Opportunities to understand current and future epidemics
Source: PLoS One. 2025 Jul 23;20(7):e0317065. doi: 10.1371/journal.pone.0317065 (PMC12286355; doi:10.1371/journal.pone.0317065)
Supplement: S1 Table — (DOCX) [file pone.0317065.s001.docx]

**Supplementary Table 1. IDCRP Acute Respiratory Infection Study Participant Information (up to n = 27,230 total participants)**

| **Study (Protocol #)** | **Age Range** | **Sex** | **Special Categories** | **Inclusion Criteria** | **Exclusion Criteria** |
| --- | --- | --- | --- | --- | --- |
| ARIC (IDCRP- 045) | 17-year- old military trainees; otherwise 18-65 year old | Male or Female | Resident/ Trainee;  Active Duty Military Personnel | 1. Between 18-65 years of age (inclusive), or a 17- year-old military service member. 2. Have an acute influenza -like illness (ILI) or SARI symptomatic for ≤ 7 days. 3. Eligible for care in DOD facilities (DEERS eligible). | No exclusion criteria has been added to this protocol. |
| H1N1 Vaccine (IDCRP-053) | 18-50 year old | Male or Female | Military Health System Beneficiary | 1. 18-50 Years of age 2. Receiving the novel H1N1 vaccine (killed formulation) as part of routine clinical care. 3. A military beneficiary who expects to remain in the local area for the next 6 months. | 1. Are a healthcare worker who sees patients (healthcare workers may be particularly likely to be exposed to influenza as part of their work and have altered immune responses due to natural exposures) 2. Have had an acute febrile illness within 30 days prior to H1N1 vaccination (e.g., pneumonia, influenza) 3. Have any of the following medical conditions: Diabetes (type 1 or type 2); Systemic steroid or immunosuppressive medication use within past 4 weeks; Active diagnoses of a cancer (non-melanoma skin cancer allowed); History of organ transplant; Chronic active hepatitis B or hepatitis C 4. Actively use illicit drug use or abuse alcohol 5. Have had a blood transfusion within last year 6. Are allergic to eggs 7. Have had a previous significant adverse reaction to the influenza vaccination |
| **EPICC (IDCRP-085)** | Any age | Male or Female | Minors/ Children; Active Duty Military Personnel; Persons with Impaired Decisional Capacity; Pregnant Women, Fetuses, and Neonates | 1. MHS beneficiaries of any age who meet at least one of the following criteria: **(a)** Laboratory-confirmed presence of the pathogen of interest; **(b)** Meet criteria for testing for the pathogen of interest, as identified per current CDC guidelines; **(c)** Received vaccine for the pathogen of interest. 2. Additional Inclusion criteria for Online Enrollment: **(a)** Able to receive email communications and respond to web-based questionnaires; **(b)** Greater than or equal to 18 years of age. | 1. Individuals who decline participation in the study. 2. Individuals who the study investigators believe are unable to comply with the requirements of the study |
| PAIVED (IDCRP- 120) | ≥ 18 years | Male or Female | Students; Resident/ Trainee; Cadets/ Midshipmen; Active Duty Military Personnel | 1. Eligible for care in DoD Facilities (DEERS eligible) 2. Greater than or equal to 18 years of age 3. At a participating MTF site for the purpose of receiving a seasonal influenza vaccination 4. Able to speak English and able to provide informed consent 5. Able to receive and respond to texts and/or emails, or a military recruit. | 1. Adults intending to receive or who have received an influenza vaccine within the current season 2. Individual who cannot receive a flu vaccine or standard dosing due to another medical condition. 3. Allergic to gentamicin, polymyxin, and/or neomycin. 4. Individuals who fail to meet the inclusion criteria. |
| CAMP NYC (IDCRP-125) | ≥ 18 years old | Male or Female | Active Duty Military Personnel | 1. Cohort 1: All Active Duty Army personnel deployed to NYC from Fort Sam Houston (San Antonio, TX), Fort Campbell, KY, and Fort Hood, TX; 2. Cohort 2: Individuals admitted to the Javits Convention Center with nasal swab PCR proven COVID-19 infection. | 1. Cohort 1: Personnel deployed from areas with high rates of community spread of COVID-19 at the time of their arrival to NYC. 2. Cohort 2: Patients without a confirmed COVID-19 positive test 3. Cohort 2: Symptom onset < 4 days prior 4. Cohort 2: Patients who are not fluent in English 5. Cohort 2: Altered mental status, dementia, or impaired cognition (as assessed by the study investigator or indicated in their medical records) 6. Cohort 2: Age less than 18 (also makes them ineligible for admission to the Javits Convention Center. |
| PASS (IDCRP- 126) | ≥ 18 years old | Male or Female | Employees- Civilian; Employees- Contractor; Resident/ Trainee; Active Duty Military Personnel | 1. Age, greater than or equal to 18 years old. 2. Generally healthy 3. Health care providers (nurses, doctors, physician assistants, respiratory therapists, occupational therapists, and medical technicians) at WRNMMC 4. Willingness and ability to return for scheduled follow-up visits, with assurance of follow-up at least for the first three months. | 1. Immunocompromised state or immune modulating medications - presence of a disease that is actively causing severe immune suppression including 20 mg prednisone or greater daily for over one month, chemotherapy, cytokine inhibitors, or agents that reduce T cell or B cell numbers or function. 2. Symptoms of fever, cough, anorexia, myalgias, chills, shortness of breath, anosmia, sore throat, rhinorrhea, or diarrhea at enrollment. 3. Presence of fever (T > 100.4 °F) on screening vital signs. 4. Known prior diagnosis with COVID-19. 5. Positive SARS-CoV-2 IgG by multiplex coronavirus serology assay. 6. Participation in a COVID-19 vaccine or prophylactic antibody study. |
| COMFORT (IDCRP-128) | ≥ 18 years old | Male/ Female | Active Duty Military Personnel | 1. Active-Duty US Navy Active-Duty and Reserve personnel participating in the COVID-19 related missions on the USNS COMFORT hospital ship. 2. Willing to provide informed consent and comply with study procedures (questionnaire and blood draw) | No exclusion criteria has been added to this protocol. |
| TOSCANA (IDCRP-129) | 17-27 | Male or Female | Cadets/ Midshipmen | 1. US Naval Academy Midshipman, male or female subject, 17-27 years of age, inclusive, at the time of screening. 2. Able to view video link advertising the study and to read and complete on-line informed consent document. 3. Informed of the nature of the study and has agreed to and is able to read, review, and sign the informed consent document prior to screening. 4. Free of known significant health problems as established by the requirements to be enrolled in a military service academy before entering into the study | 1. Not able to view video link advertising the study and to read and complete on-line informed consent document. 2. Individuals who fail to meet the inclusion criteria. |
| PISCES (IDCRP-  130) | Children:  > 2-17 years;  Adults:  ≥18 years | Male or Female | Minors/Children; Students; Employees – civilians; Employees – contractors; Resident/Trainee; Active Duty Military Personnel; Pregnant  Women,  Fetuses, and  Neonates | 1. USU affiliate: male or female 18 years of age (primary enrollee) 2. USU affiliate: A USU faculty, staff, student, Active-Duty military, or with the USU Bethesda, MD campus as their primary place of duty outside the home and current functioning usuhs.edu email address. 3. Household member of the primary enrollee, >24 months of age, or parent or guardian of the individual is 18 years of age. 4. Volunteer or parent/legal guardian able to a video link advertising the study and to read and complete on-line informed consent document. 5. Volunteer or parent/guardian informed of the nature of the study and has agreed to and is able to read, review, and sign the informed consent document prior to screening. | 1. Volunteer or parent/legal guardian not able to view video link advertising the study and to read and complete on-line informed consent document. 2. Current participation in COVID-19 vaccine trials or any prospective studies that includes investigational pre- or post- exposure prophylaxis products. Future vaccine receipt or receipt of any prophylaxis product will not be grounds for removal from the study. 3. < 24 month of age household member |

CDC = Centers for Disease Control & Prevention; DoD = Department of Defense; DEERS = Defense Enrollment Eligibility Reporting System; WRNMMC = Walter Reed National Military Medical Center; MTF = Military Treatment Facility
